# Obstructive sleep apnoea accelerates FEV_1_ decline in asthmatic patients

**DOI:** 10.1186/s12890-017-0398-2

**Published:** 2017-03-21

**Authors:** Tsai-Yu Wang, Yu-Lun Lo, Shu-Min Lin, Chien-Da Huang, Fu-Tasi Chung, Horng-Chyuan Lin, Chun-Hua Wang, Han-Pin Kuo

**Affiliations:** 1 Pulmonary Disease Research Centre, Department of Thoracic Medicine, Chang Gung Memorial Hospital, Chang Gung University, School of Medicine, 199 Tun-Hwa N. Rd., Taipei, Taiwan; 2 Department of Thoracic Medicine, St. Paul’s Hospital, Taoyuan, Taiwan

**Keywords:** Obstructive sleep apnoea, Pulmonary function, asthma

## Abstract

**Background:**

Although the prevalence of both obstructive sleep apnoea (OSA) and asthma are both increasing, little is known about the impact of OSA on the natural history of lung function in asthmatic patients.

**Methods:**

A total of 466 patients from our sleep laboratory were retrospectively enrolled. Of them, 77 patients (16.5%) had asthma with regular follow-up for more than 5 years. Their clinical characteristics, pulmonary function, emergency room visits, and results of polysomnography results were analysed.

**Results:**

The patients were divided into three groups according to the severity of the apnoea-hypopnea index (AHI). The decline in FEV_1_ among asthma patients with severe OSA (AHI > 30/h) was 72.4 ± 61.7 ml/year (*N* = 34), as compared to 41.9 ± 45.3 ml/year (*N* = 33, *P* = 0.020) in those with mild to moderate OSA (5 < AHI ≤ 30) and 24.3 ± 27.5 ml/year (*N* = 10, *P* = 0.016) in those without OSA (AHI ≤ 5). For those patients with severe OSA, the decline of FEV_1_ significantly decreased after continuous positive airway pressure (CPAP) treatment. After multivariate stepwise linear regression analysis, only AHI was remained independent factor for the decline of FEV_1_ decline.

**Conclusions:**

Asthmatic patients with OSA had substantially greater declines in FEV_1_ than those without OSA. Moreover, CPAP treatment alleviated the decline of FEV_1_ in asthma patients with severe OSA.

## Background

Although both asthma and obstructive sleep apnoea (OSA) are common diseases with increasing prevalence, the impact of OSA on the natural history of pulmonary function decline has not been well described. Increased mortality and morbidities in those with declining pulmonary function have been revealed in previous studies on asthmatic patients selected from the general population [1–3]. These highlight the role of the decline rate in pulmonary function in asthmatic patients. Factors associated with such rapid decline of pulmonary function include age, sex, smoking, acute exacerbation, obesity, and hypoxia [4–6]. While obesity is associated with OSA, hypoxia during sleep is also a cardinal feature of OSA [7]. As previous reports, both obesity and hypoxia aggravated the decline of pulmonary function, which implies the role of OSA in pulmonary function decline of asthmatic patients. In addition, the impact of OSA on asthma control such as symptoms, peak flow rate, acute exacerbation and quality of life are well documented [7–11]. Moreover, acute exacerbation which is related to the decline of pulmonary function in asthmatic patients can be reversed by continuous positive airway pressure (CPAP) [10, 12, 13]. Therefore, we hypothesize that OSA is an independent factor associated with the decline in pulmonary function in patients with asthma, and CPAP treatment prevents the decline by improving nocturnal hypoxia and frequency of acute exacerbation.

Although previous studies show that CPAP treatment cannot improve pulmonary function in asthmatic patients with OSA [10, 12], the impact of OSA on pulmonary function decline may require long-term longitudinal follow-up studies. In this study, the aim of this study was to evaluate the annual pulmonary function decline in asthmatic patients with OSA in comparison to those without in a sleep laboratory-based population, and to examine the clinical efficacy of CPAP treatment. The analyses were mainly based on asthmatic patients with more than 5 years of follow-up in Chang Gung Memorial Hospital, Linko and Taipei, Taiwan.

## Methods

### Study population

From January to August 2011, asthmatic patients were retrospectively recruited from the sleep laboratory of Chang Gung Memorial Hospital, a tertiary hospital in Taiwan. Patients were excluded if they were not regularly followed-up more than 5 years. The Chang Gung Medical Foundation Institutional Review Board (103-1609B) approved the study and waived the requirement for informed consent due to the retrospective nature of this study.

### Study design

Each patient’s medical records were reviewed to collect the clinical characteristics and laboratory results. Information, including results of pulmonary function, polysomnography, and emergency department (ER) visits due to exacerbations of asthma and CPAP compliance, were analysed. For asthmatic patients with severe OSA, another 2-year follow-up for pulmonary function test were also collected for further analysis. Pulmonary function such as FEV_1_ and FVC were measured by an electronic spirometer, which was calibrated daily using a 1-litre syringe. For the correct performance of the procedure as standard requirement, the difference between at least two FEV_1_ measurements was <5%. In addition, the highest value of FEV_1_ was chosen for the analyses.

### Definitions

All patients reported a personal history of asthma such as episodic breathlessness, cough, wheezing, chest tightness, and seasonal variability. Asthma was confirmed by clinical and functional assessment as defined by the American Thoracic Society criteria [14]. Airway reversibility was defined as 12% and 200 ml increase in FEV_1_ or average daily diurnal peak flow variability is more than 10% [15]. Regular follow-up was defined as a return to outpatient clinics of at least every 3 months with pulmonary function test at least every 6 months. Emergency room visits were considered when patients went to the ER due to asthma exacerbation. A maximum of one ER visit every 3 months was counted. From the charts, the patients reported themselves as current smokers, ex-smokers, or never-smokers, but both ex-smokers and never-smokers were defined as non-smokers.

### Polysomnography and CPAP titration

Polysomnography (Alice 5, Respironics) was performed on all patients using standard techniques. Sleep stages and arousals were scored according to the AASM criteria [16]. Established criteria were used to score respiratory events such as hypopnea, obstructive apnoea, central apnoea, mixed type apnoea, and Cheyne-Stokes respiration [17, 18]. Apnoea was defined as oronasal flow cessation for more than 10 s. Hypopnea was defined as a 50% reduction in oronasal flow or a 30% reduction, followed by arousal or more than 3% decrease in SaO2 [17].

Based on the polysomnography results, OSA was defined as an apnoea-hypopnea index (AHI) >5 per hour, of which ≥80% were obstructive. Mild-to-moderate OSA was defined as AHI >5 per hour and AHI ≤30 per hour. Severe OSA was defined as AHI >30 per hour. To determine optimal pressure, CPAP titration was performed according to standard guidelines [19]. Good CPAP compliance was defined as >4 h per day for >5 days per week.

### Statistical analysis

Data were expressed as mean ± SEM (standard error of the mean). One-way ANOVA was used for comparison of continuous variables among the three groups, while the Kruskal-Wallis test was used for non-normal distributions. Categorical variables were compared by *χ*2 or Fisher’s exact test. The Pearson product correlation coefficient was used to examine correlations between variables and the decline in FEV_1_. Multivariate stepwise linear regression analysis was used to determine independent factors affecting the decline in FEV_1_. Statistical significance was set at *p* **<** 0.05. Analysis conducted using the SPSS (version 13.0; SPSS; Chicago, IL) statistical software.

## Results

### Subject characteristics and polysomnography results

There were 466 patients with polysomnography results from the sleep laboratory, including 77 (16.5%) with asthma and regular follow-up more than 5 years. Their baseline demographics and clinical characteristics (Table 1) revealed that the mean age of asthmatic patients without OSA, those with mild-to-moderate OSA, and those with severe OSA was 49.0 years, 60.0 years, and 62.9 years, respectively. The other characteristics were similar among the three groups, including the percentage of males, and baseline pulmonary function such as FEV_1_ and FVC in the first year. From the results of polysomnography (Table 2), AHI, ODI, average SaO2, and lowest SaO2, as well as sleep architecture like slow-wave sleep stage (N3) and rapid eye movement (REM) stage, were significantly worse in asthmatic patients with severe OSA.

**Table 1:** Patient characteristics

Characteristics	No OSA	Mild-to-Moderate OSA	Severe OSA	*p* value
	*n* = 10	*n* = 33	*n* = 34	
Age	49.0 ± 13.6	60.0 ± 13.3	62.9 ± 12.7	0.021
Male, n (%)	6 (60)	25 (75.8)	20 (58.8)	0.348
BMI	23.4 ± 4.0	24.8 ± 4.3	29.7 ± 4.7	0.001
Current Smoker, n (%)	2 (20.0)	10 (30.3)	12 (35.3)	0.653
Pulmonary function Test (1st year)				
FEV_1_/FVC	71.9 ± 13.2	73.2 ± 18.9	79.1 ± 13.9	0.078
FEV_1_, Litre	2.12 ± 0.44	1.75 ± 0.82	1.91 ± 0.80	0.400
FEV_1_ (% predicted)	68.6 ± 15.9	65.3 ± 24.6	77.1 ± 25.3	0.132
FVC, Litre	2.94 ± 0.42	2.39 ± 0.83	2.41 ± 0.84	0.096
Decline of FEV_1_/year, (% from baseline)	1.3 ± 1.3	3.1 ± 3.3	4.3 ± 3.4	0.024
Decline of FEV_1_/year, (% predicted)	0.0 ± 1.1	1.0 ± 1.9	2.3 ± 3.0	0.027
Decline of FEV_1_/year (mL)	24.3 ± 27.5	41.9 ± 45.3	72.4 ± 61.7	0.046
Decline of FVC/year, (% from baseline)	1.1 ± 3.6	1.4 ± 3.4	2.6 ± 3.6	0.499
Decline of FVC/year, (% predicted)	0.6 ± 3.4	0.8 ± 2.6	1.6 ± 2.9	0.608
Decline of FVC/year, (mL)	32.5 ± 111.1	39.9 ± 84.4	54.9 ± 76.1	0.735
ER visit, n/year	0.11 ± 0.14	0.48 ± 0.63	0.52 ± 0.62	0.256
Medication				
Inhaled glucocorticoid	9 (90)	33 (100)	31 (91.2)	0.208
Inhaled long-acting beta-agonist	6 (60)	25 (75.8)	30 (88.2)	0.128
Leukotriene antagonist	1 (10)	10 (30.3)	21 (61.8)	0.003

Data are presented as mean ± SEM, or number (percentage)

Abbreviations: *OSA* obstructive sleep apnoea, *BMI* body mass index, *FEV*_1_ forced expiratory volume in 1 second, *FVC* forced volume capacity, *ER* emergency department

**Table 2:** Polysomnography results

Characteristics	No OSA	Mild-Moderate OSA	Severe OSA	*p* value
	*n* = 10	*n* = 33	*n* = 34	
Total sleep time (minutes)	288.2 ± 88.2	268.3 ± 80.7	253.8 ± 74.6	0.207
Sleep efficiency (%)	76.1 ± 22.9	75.8 ± 16.1	72.1 ± 18.0	0.506
AHI event. h^−1^	2.4 ± 1.5	16.2 ± 6.6	61.4 ± 20.0	0.001
ODI event. h^−1^	1.7 ± 1.7	14.5 ± 6.1	52.1 ± 22.8	0.001
Average SaO_2_ (%)	95.0 ± 1.3	93.1 ± 3.0	91.4 ± 3.3	0.001
Lowest SaO_2_ (%)	88.2 ± 6.0	82.2 ± 5.5	74.7 ± 9.7	0.001
SaO2 < 90% (%)	0 ± 0	9.1 ± 18.2	20.5 ± 25.9	0.001
SaO2 < 80% (%)	0 ± 0	0.2 ± 0.4	3.3 ± 5.2	0.021
Wake %	13.9 ± 8.1	22.4 ± 13.7	22.7 ± 17.8	0.226
N1 %	20.8 ± 11.0	16.5 ± 11.0	22.3 ± 10.7	0.423
N2 %	27.6 ± 11.5	28.8 ± 11.2	34.1 ± 14.1	0.125
N3 %	23.6 ± 8.4	22.1 ± 10.8	13.4 ± 9.8	0.001
REM %	14.1 ± 3.4	10.3 ± 8.5	7.4 ± 5.9	0.013

Data are presented as mean ± SEM, or number (percentage)

Abbreviations: *OSA* obstructive sleep apnoea, *AHI* apnoea-hypopnoea index, *ODI* oxygen desaturation index, *SWS* slow wave sleep, *REM* rapid eye movement, *CPAP* continuous positive airway pressure

### Annual decline of FEV_1_ in asthmatic patients with different OSA severities

**Fig. 1:**
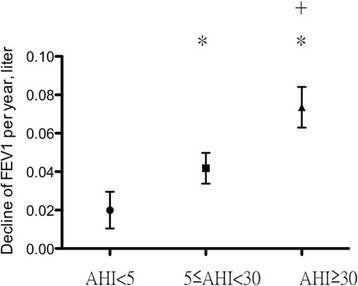
Decline in FEV_1_ per year among the three groups. AHI, apnoea-hypopnoea index; *p < 0.05 compared to AHI <5; ^+^ *p* < 0.05 compared to 5 ≤ AHI < 30

The most important finding of the present study was that the annual decline in FEV_1_ of asthmatic patients with severe OSA was significantly accelerated compared to those of patients with mild-to-moderate OSA and those of patients without OSA (72.4 ± 61.7 milli-litre vs. 41.9 ± 45.3 milli-litre vs. 24.3 ± 27.5 milli-litre, *p* = 0.046) (Fig. 1). In contrast, the annual decline in FVC was not significantly different among the three groups.

**Fig. 2:**
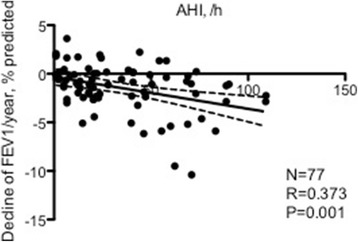
Correlation between the apnoea-hypopnoea index (AHI) and the decline of FEV_1_ per year, % predicted

In terms of possible aetiologies responsible for the annual decline of FEV_1_, % from baseline, univariate analysis (Tables 3) revealed that age, AHI, ODI, and ER visit were significantly associated with the annual decline in FEV_1_, % predicted (Fig. 2). Although BMI was significantly different between the three groups, it was not significantly associated with the annual decline in FEV_1_, % from baseline. After multivariate stepwise linear analysis, only AHI remained independent factor associated with the annual decline in FEV_1_, % predicted (Table 4).

**Table 3:** Univariate analysis of variables associated with average decline of FEV_1_, % predicted

Parameter	beta	Standard error	95% CI	*p* value
Age	−0.053	0.020	−0.093 to − 0.012	0.011
ESS	−0.280	0.085	−0.323 to 0.021	0.084
BMI	−0.077	0.056	−0.189 to 0.036	0.178
Current smoke	−1.173	0.599	−2.367 to 0.021	0.054
AHI	−0.033	0.009	−0.052 to − 0.014	0.001
ODI	−0.035	0.010	−0.056 to −0.015	0.001
SaO2 < 90% (%)	−0.014	0.015	−0.043 to 0.015	0.347
SaO2 < 80% (%)	−0.091	0.094	−0.280 to 0.097	0.336
ICS	−0.091	1.646	−4.581 to 1.977	0.432
ER visit, n/year	−0.941	0.470	−1.877 to − 0.005	0.049

Abbreviations: *ESS* epworth sleepiness scores, *BMI* body mass index, *AHI* apnoea-hypopnoea index, *ODI* oxygen desaturation index, *ICS* inhaled corticosteroid, *ER* emergency department

**Table 4:** Multivariate stepwise analysis of variables associated with the average decline of FEV1, % predicted

Parameter	beta	Standard error	95% CI	*p* value
AHI	−0.033	0.009	−0.052 to − 0.014	0.001

Abbreviations: *AHI* apnoea-hypopnoea index, *ODI* oxygen desaturation index, *ER* emergency department

### CPAP treatment alleviated the rapid decline of FEV_1_ in asthmatic patients with severe OSA

**Fig. 3:**
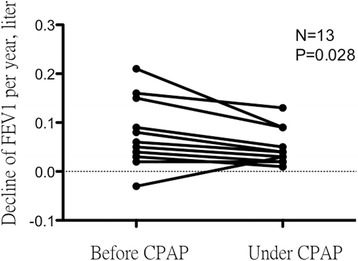
Comparison of the decline in FEV_1_ per year among asthmatic patients with severe obstructive sleep apnea before and after CPAP treatment

Thirty-eight percent (13/34) of asthmatic patients with severe OSA treated with CPAP had good compliance. In the annual decline in FEV_1_ before and after CPAP treatment (Fig. 3), after adequate CPAP treatment for the next 2 years, the annual decline in FEV_1_ was 41.2 ± 36.1 mL, which was significantly lower than that before CPAP treatment (69.4 ± 66.4 mL, *p* = 0.028). The frequency of ER visits were also decreased after CPAP treatment from 0.35 ± 0.52 per year to 0.35 ± 0.52 per year but there was just trend (*P* = 0.058). The average CPAP pressure was 8.9 ± 4.2 cmH_2_O and the average time of CPAP use was 6.4 h per night.

## Discussion

The present study demonstrates that asthmatic patients with obstructive sleep apnoea have a greater decline in FEV_1_ over time than those without OSA. Although age, AHI, ODI, and ER visits are significant factors associated with a greater decline in FEV_1_ under univariate analysis, only AHI is an independent factor in multivariate analysis. Moreover, CPAP treatment alleviates the accelerated decline in FEV_1_ in asthmatic patients with severe OSA, which further consolidates the role of OSA in the decline in FEV_1_ among asthmatic patients.

The natural history of FEV_1_ decline in asthmatic patients has been reported to be 38–40.9 ml/year [6, 20–22]. Factors associated with the decline in pulmonary function are age, sex, smoking, acute exacerbation, obesity, and hypoxia [4–6]. Furthermore, OSA with obesity and hypoxia as central features, significantly contribute to asthma control and exacerbation. Thus, it is not surprising that CPAP treatment of up to 2 years for asthmatic patients with severe OSA is beneficial not only in terms of quality of life and in alleviating asthma symptoms but also in reducing FEV_1_ decline.

Frequent symptoms and exacerbations have been reported to be associated with an excess decline in lung function among asthmatics [23, 24]. Similarly, the results of present study also reveal that the number of ER visits is associated the decline in FEV_1_. For asthma control, the presence of OSA is associated with more symptoms, exacerbations and worse quality of life. Furthermore, CPAP treatment can alleviate the symptoms, the frequency of exacerbations and improve the quality of life [9–12]. Therefore, the decline of FEV_1_ may be alleviated by CPAP treatment in asthmatic patients with OSA. This is the first study reporting more exacerbations in asthmatic patients with OSA than in patients without OSA based on sleep lab population. In addition, CPAP treatment can reduce exacerbations in asthmatic patients with OSA. Such exacerbation is also associated with the decline in pulmonary function among asthmatic patients with OSA.

Inhaled corticosteroid (ICS) can alleviate the decline of FEV_1_ [22]. In addition, asthmatic patients with obesity are poor response to ICS [25]. The National Heart Lung and Blood Institute–sponsored Severe Asthma Research Program (SARP) has identified and characterized a phenotype of severe asthma consisting mainly of non-atopic late-onset older women with the highest body mass index, who are poor responders to ICS and frequently require oral corticosteroid use to manage exacerbations (Cluster 3). [26] Obesity is frequently associated with OSA. Therefore, asthmatic patients with OSA may also be poor responders to ICS, leading to accelerate the decline of FEV_1_. Another possible reason responsible to FEV_1_ decline due to obesity is that obesity may reduce FEV_1_ and FVC concurrently. However, this study reveals that the baseline FEV_1_ is not significantly different between groups and there is trend in FVC, which is worse in severe OSA group. Therefore, obesity did not reduce FEV_1_ and FVC concurrently in this study. Furthermore, BMI is not significant associated with the decline of FEV_1_ in univariate analysis. Therefore, obesity is not a significant factor associated with the decline of FEV_1_ in asthmatic patients with OSA.

The major limitations of the present study include its retrospective nature, which may lead to bias in patient selection, and a relatively small sample size that may lead to spurious associations and conclusions. Pro-inflammatory profiles of asthma, such as FeNO, sputum or peripheral eosinophil counts were not entirely measured in every study subject in this retrospective study. A long-term, prospective study with a larger scale study is needed to corroborate these findings, and include the pro-inflammatory profiles into analysis for the association with annual decline in pulmonary function.

## Conclusions

In conclusion, in a sample of the sleep-laboratory population, asthmatic patients with OSA have a substantially greater decline in FEV_1_ in 5-year follow-up compared to those without OSA. The impact of OSA on the annual decline in FEV_1_ is dose dependent. Moreover, CPAP treatment alleviates the decline in FEV_1_ among asthmatic patients with severe OSA.

## Abbreviations

AHI: Apnoea-hypopnoea index
BMI: Body mass index
CPAP: Continuous positive airway pressure
ER: Emergency department
FEV_1_: Forced expiratory volume in 1 second
FVC: Forced vital capacity
ODI: Oxygenation desaturation index
OSA: Obstructive sleep apnoea
REM: Rapid eye movement
